# Unilateral Anophthalmic Socket Reconstruction With Dermis Fat Graft

**DOI:** 10.7759/cureus.18834

**Published:** 2021-10-17

**Authors:** Saba Alkhairy, Mahad M Baig, Usman A Pasha

**Affiliations:** 1 Ophthalmology, Dow University of Health Sciences, Dow International Medical College, Karachi, PAK

**Keywords:** isolated anophthalmia, decreased orbital volume, unilateral anophthalmia, orbital volume, prosthetic eye, conjunctival insufficiency, reconstruction, cosmetic, mucous membrane graft, dermis fat graft

## Abstract

A 15-year-old girl presented with the complaint of a cosmetically disfigured right eye since birth. The initial assessment was carried out by way of a history and physical examination. The patient’s history revealed no significant findings. Physical examination revealed that the right ophthalmic socket was seemingly devoid of an identifiable globe and ocular tissue, which is described as an anophthalmic socket. The conjunctival fornices appeared short, a finding which was more noticeable in the inferior conjunctival fornix. The patient’s orbital volume was decreased. To increase the orbital volume, a dermis fat graft (DFG) was carefully taken from the gluteal region. The graft was then transposed to the anophthalmic socket and sutured to the conjunctiva and Tenon’s capsule. A prosthetic eye was placed in the socket. Later, a second surgical intervention was performed to deepen the inferior fornix, for which a mucous membrane graft was taken from the lower lip. As a result of these interventions, all cosmetic and medical concerns of the patient regarding the anophthalmic socket were addressed. The success of this procedure speaks to the efficacy of DFGs in the repair of an anophthalmic socket.

## Introduction

Anophthalmia (AO) is a rarely occurring birth defect characterized by the complete absence of the eye [1]. Microphthalmia (MO) is a condition which has frequently been cited in the same context as anophthalmia. In contrast to anophthalmia, microphthalmia refers to the presence of a miniature eye in the orbit [2]. Often, the terms are used interchangeably, as the clinical basis for diagnosis is formed on findings which can be expressed on a spectrum from MO to AO [2]. Although both microphthalmia and anophthalmia may manifest unilaterally or bilaterally, unilateral AO is rarer than its bilateral counterpart (with the exception of isolated bilateral MO) [3]. 

The hypothesis which attempts to explain the pathogenesis of this manifestation asserts either that this abnormality is a result of a developmental failure of the anterior neural tube or that it is due to the growth failure of the optic pit(s) [4]. From this explanation, anophthalmia may be classified as primary or secondary. Therein, primary AO refers to the developmental failure of the anterior neural tube, whereas secondary AO refers to the developmental failure of the optic pit(s). However, this does not fully explain the third classification of AO, which is termed as consecutive AO (also known as degenerative AO). This classification identifies cases of AO which present evidence of degenerated optic vesicles [5]. 

Presentation-wise, neonates born with AO may possess an empty orbital socket, shortened eyelids, and reduced orbital volume. Additionally, in about one-third of cases, AO/MO may present as a component of a comorbid syndrome including Curry-Jones syndrome, Waardenburg syndrome, or Matthew-Wood syndrome [2,6]. Such cases are deemed as complex AO/MO. Other etiologies include genetic defects, chromosomal aberrations, radiation exposure, viral infections, teratogenic agents, or increased maternal age at birth of the patient [7]. 

Anophthalmia can be diagnosed either prenatally or postnatally. Prenatal diagnosis can be achieved through the use of an ultrasound scan to assess the contents of the fetal orbit, whereas postnatal diagnosis usually involves a thorough physical examination of the eye and possibly an MRI/CT scan in order to assess the potentially involved ocular structures [8]. Genetic tests may be administered in the case that a genetic syndrome is suspected. Genetic syndromes may be considered in the case that a pathognomonic constellation of symptoms is presented or if similar anomalies exist within the family [8].

Although this congenital disorder is incurable, surgical procedures may be performed in order to accomplish AO socket reconstruction for remedying facial disfigurement. For this purpose, a prosthetic eye may be interposed within the socket, and a dermis fat graft (DFG) may be done with the aim of increasing orbital volume [2]. 

## Case presentation

The patient was received in the Eye Out-Patient Department (OPD) of Dow University of Health Sciences (DUHS), in Karachi, Pakistan, in December 2018. The patient was accompanied by her biological mother. She identifies herself as female and reports that she is 14-years-old. The patient presents with complaints of a cosmetically disfigured right eye (OD) since birth, which is attested to by the mother. 

Upon continuation of the history-taking interview, no significant findings which would have indicated exposure to risk factors and known etiologies were revealed. This aspect of the history was taken with the cooperation of the mother in order to receive an account of the patient’s fetal development. The patient’s mother therein denied any exposure to radiation, trauma, or drugs during pregnancy. Furthermore, the patient was reported to have been delivered via uncomplicated vaginal delivery to non-consanguineous parents which occurred at term. There was no history of maternal fever, rash, or jaundice throughout the entirety of pregnancy. The patient herself reported no significant history of trauma, ocular tumor, radiation exposure, nor endophthalmitis, which was corroborated by the mother. Finally, inquiry of the patient's family history, regarding birth defects and congenital anomalies, revealed no significant findings; neither did a review of systems yield anything of concern. 

Upon physical examination of the eye, It was confirmed that the right ophthalmic socket was entirely devoid of a globe and identifiable ocular tissue. The conjunctival fornices of the right eye were shortened, especially the inferior fornix, as measured using a conjunctival depth measurer (CDM). OD visual acuity was absent. The orbital volume appeared reduced. Despite these anomalies, the patient’s lid margins, eyelashes, and levator palpebrae function were found to be within tolerable limits. The left eye was clinically insignificant for any physical findings, and visual acuity was within normal limits. A general physical examination was clear of any abnormalities. These findings conform to the clinical diagnosis of unilateral, congenital, isolated right anophthalmic socket. The patient and family subsequently provided informed consent for surgical intervention and eventual placement of a prosthesis in order to address the cosmetic disfigurement. 

**Figure 1:**
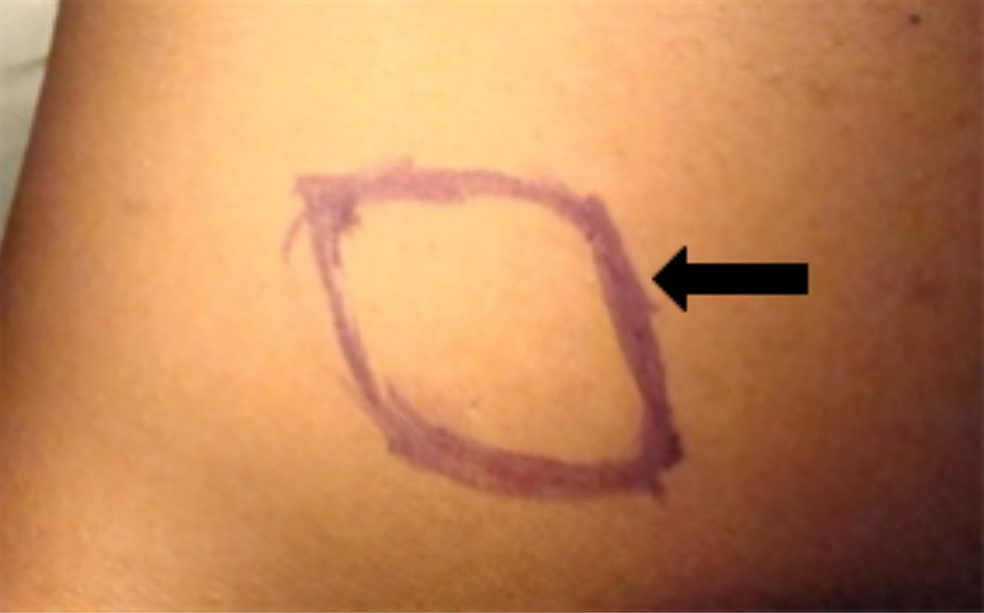
Dermis fat graft incision outline

**Figure 2:**
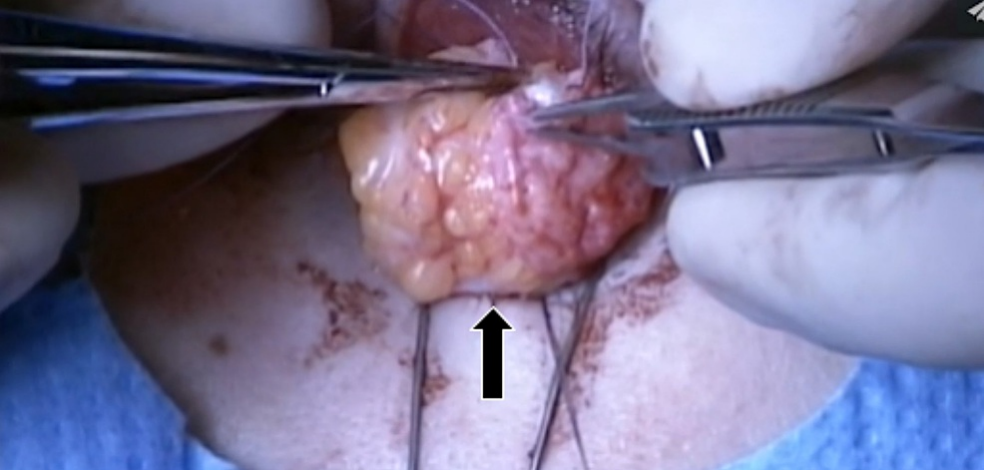
Dermis fat graft being placed in the anophthalmic socket

The first in what would become a two-part series of operations were performed for the purpose of increasing the orbital volume. To accomplish this, a dermis fat graft (DFG) was harvested from the superior-lateral portion of the left gluteal region after being outlined using a marker (Figure 1). The incision was about 30% larger than the area of the orbital defect. This DFG was then placed into the prepared AO socket. A single interrupted 6-0 polyglactin suture was placed in order to stitch the graft to the Tenon’s capsule and conjunctiva (Figure 2). Subsequently, a conformer was installed temporarily to prevent friction secondary to the movement of the eyelid over the suture lines. This device also serves to maintain the shape of the socket until it is ready for the prosthetic. Tarsorrhaphy was performed in order to reduce the risk of infection. 

**Figure 3:**
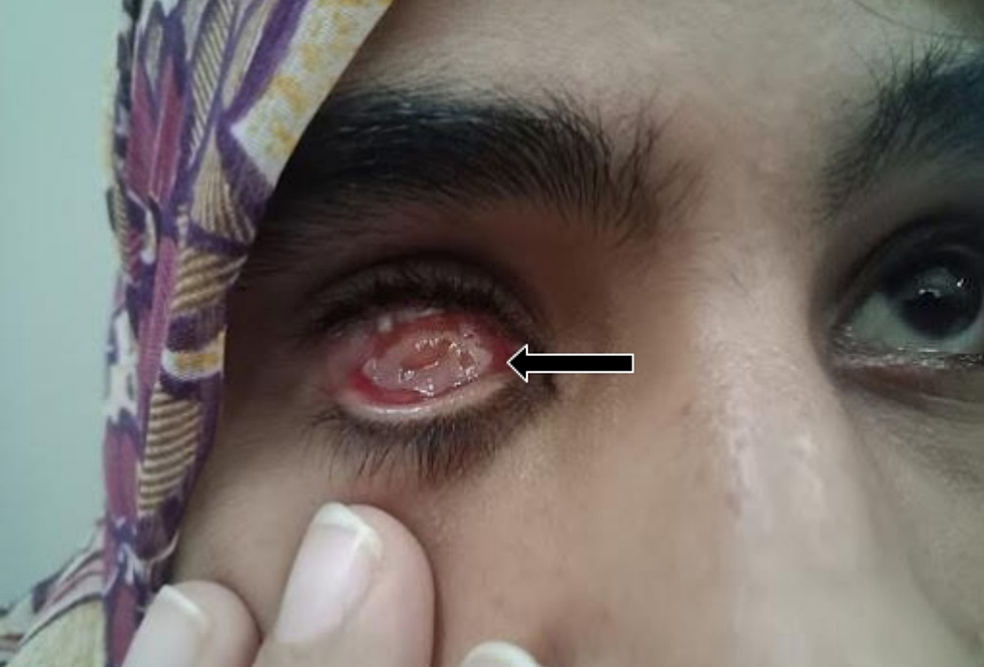
Patient follow-up of dermis fat graft

**Figure 4:**
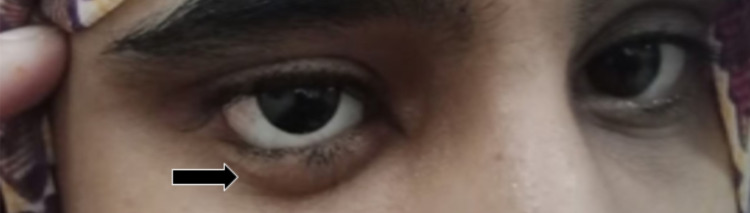
Cosmetically undesirable bulge under the lower lid due to conjunctival insufficiency

At a six-week follow-up appointment, the tarsorrhaphy sutures were undone, revealing unpredictable absorption of up to 70% of the DFG (Figure 3), indicating a successful transplant. The socket was thereafter ready to receive the prosthesis. Unfortunately, a cosmetically undesirable bulge was discovered upon placement of the artificial eye, as observed within the lower eyelid (Figure 4). Hence, a second surgery was undertaken to deepen the conjunctival fornix through the use of a mucous membrane graft, which was harvested from the lower lip and stitched to the inferior conjunctiva using a similar 6-0 proteoglycan suture. 

**Figure 5:**
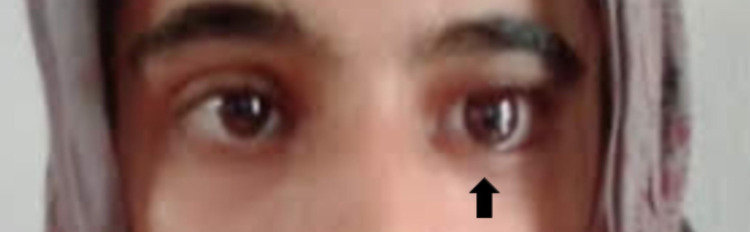
Final result of anophthalmic socket reconstruction surgery

Upon follow-up of the second procedure, the implant was fitted successfully; it presented free of cosmetic defects (Figure 5). Hence, the AO socket was successfully reconstructed and fitted with a prosthetic eye without cosmetic disfigurement. The patient expressed great satisfaction with the final result and exhibited increased self-esteem, to the joy of her family.

## Discussion

The cosmetic disfigurement associated with anophthalmia gives rise to both social and emotional problems. The correction of this disfigurement is essential for patients to more easily live their lives, free of the consequences of societal judgment. For this issue to be addressed by the use of a prosthetic eye, patients must often, first, undergo treatment to resolve pre-existing reduced orbital volume, or the so-called “sunken appearance” of the orbit. This is frequently the primary obstacle obstructing the placement of the prosthetic implant. In order to increase the orbital volume, either dermis fat grafts (DFGs) or alloplastic grafts are often used. DFGs are taken from human or animal sources, while alloplastic grafts are taken from naturally occurring elements or synthetic materials. Although alloplastic grafts have been used historically with some efficacy, a growing body of scientific literature shows that autologous DFGs are an exceedingly safe option in comparison to alloplastic grafts, especially in pediatric patients and complicated cases [9]. This becomes especially apparent in light of the possible complications of allografts, including the cost of treatment, unpredictable reabsorption, adverse reactions, and unpredictable vascularization rate [10].

**Figure 6:**
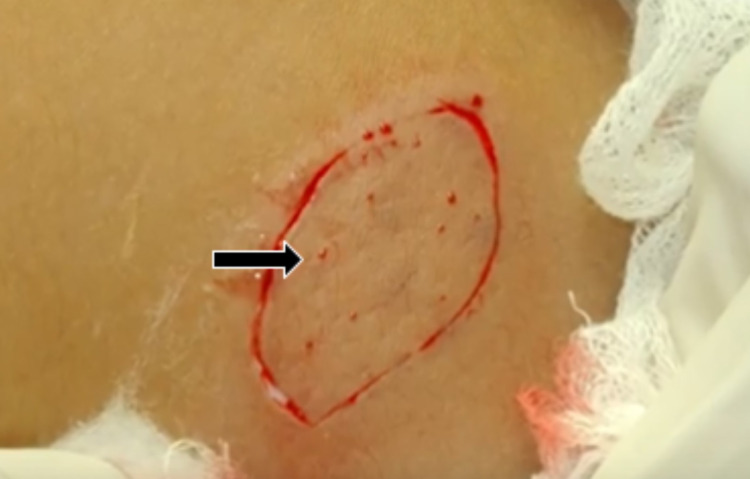
Dermis fat graft site showing petechiae

**Figure 7:**
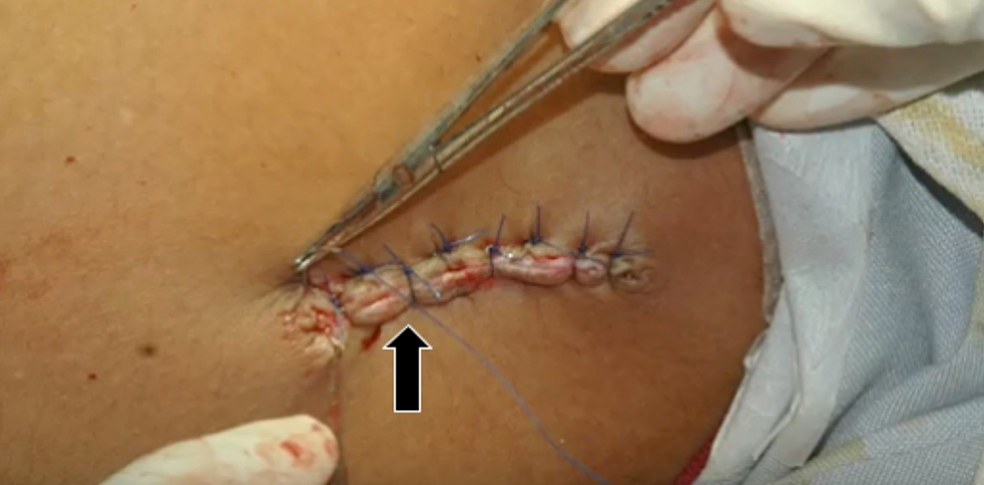
Dermis fat graft wound closure

DFGs can be harvested from the upper lateral portion of the patient’s own gluteal region. A superficial incision is used to dissect the epidermis from the dermis. If performed correctly, a petechial bleeding pattern may present itself upon the surface of the dermis, as observed in this case (Figure 6). Wound closure (Figure 7) must be completed in layers in order to decrease tension and reduce the risk of breakdown. The DFG tissue is then interposed under the eyelid of concern by stitching it to the Tenon’s capsule and conjunctiva. Finally, a conformer is placed before the conclusion of the surgery. The most feared complication is graft loss second to necrosis or infection. Even so, research shows that the prevalence of such an outcome may be less than 3% [11]. 

**Figure 8:**
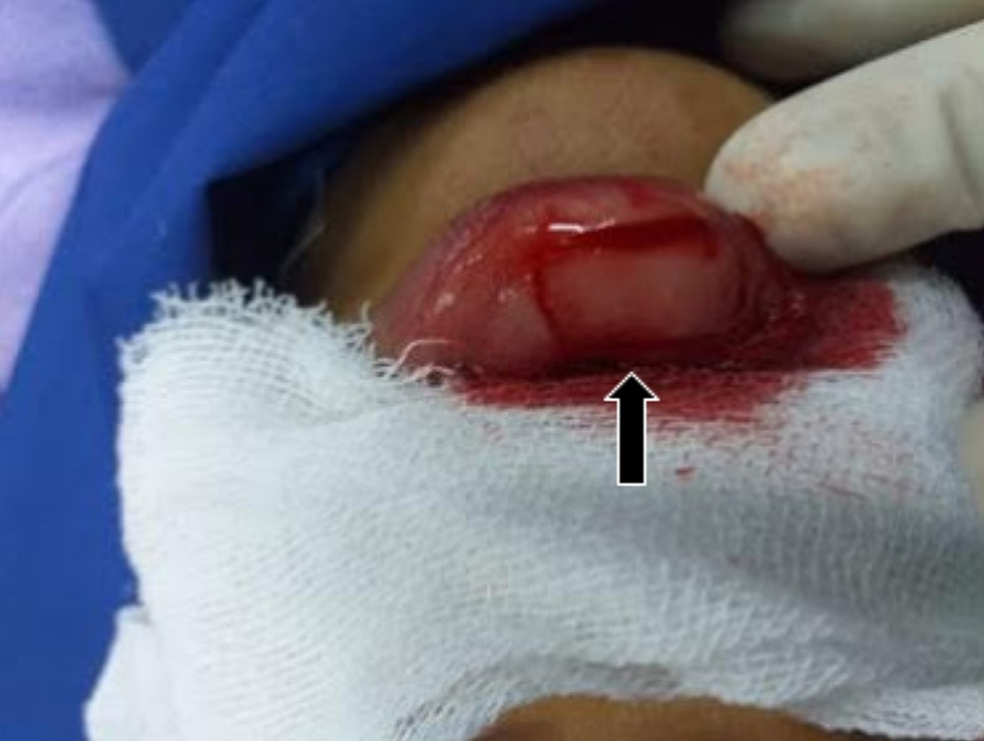
Mucous membrane graft incision site

In this case, conjunctival insufficiency was found upon the attempted placement of the prosthetic, warranting a second procedure using a mucosal transplant from the lower lip (Figure 8), which is an approach possessing demonstrable efficacy as presented by a recent literature review [12]. Although the inner cheek is usually preferred for harvesting, constraints pertaining to the circumstances of this case limited the ease and safety by which that location could be accessed. 

## Conclusions

Isolated unilateral anophthalmia is poorly elucidated, in part, as a consequence of its rarity. In fact, little recent literature is available on this topic. Thus, the extensive documentation of these cases must be continued in order to aid in the discovery of previously unknown etiologies which may improve our understanding of this condition. 

Additionally, the use of a DFG in combination with a mucous membrane graft, as detailed within this report, speaks to the efficacy of both approaches to more safely resolve the crippling cosmetic defects associated with AO/MO irrespective of lateralization or isolated manifestation. Therefore, this work adds to the developing body of literature which scrutinizes emerging surgical techniques such as the use of DFGs along with mucus membrane grafts for the purpose of obtaining a better understanding of the limits and various applications of such therapies.
